# JABAWS 2.2 distributed web services for Bioinformatics: protein disorder, conservation and RNA secondary structure

**DOI:** 10.1093/bioinformatics/bty045

**Published:** 2018-01-30

**Authors:** Peter V Troshin, James B Procter, Alexander Sherstnev, Daniel L Barton, Fábio Madeira, Geoffrey J Barton

**Affiliations:** Division of Computational Biology, School of Life Sciences, University of Dundee, Dundee, UK

## Abstract

**Summary:**

JABAWS 2.2 is a computational framework that simplifies the deployment of web services for Bioinformatics. In addition to the five multiple sequence alignment (MSA) algorithms in JABAWS 1.0, JABAWS 2.2 includes three additional MSA programs (Clustal Omega, MSAprobs, GLprobs), four protein disorder prediction methods (DisEMBL, IUPred, Ronn, GlobPlot), 18 measures of protein conservation as implemented in AACon, and RNA secondary structure prediction by the RNAalifold program. JABAWS 2.2 can be deployed on a variety of in-house or hosted systems. JABAWS 2.2 web services may be accessed from the Jalview multiple sequence analysis workbench (Version 2.8 and later), as well as directly via the JABAWS command line interface (CLI) client. JABAWS 2.2 can be deployed on a local virtual server as a Virtual Appliance (VA) or simply as a Web Application Archive (WAR) for private use. Improvements in JABAWS 2.2 also include simplified installation and a range of utility tools for usage statistics collection, and web services querying and monitoring. The JABAWS CLI client has been updated to support all the new services and allow integration of JABAWS 2.2 services into conventional scripts. A public JABAWS 2 server has been in production since December 2011 and served over 800 000 analyses for users worldwide.

**Availability and implementation:**

JABAWS 2.2 is made freely available under the Apache 2 license and can be obtained from: http://www.compbio.dundee.ac.uk/jabaws.

## 1 Introduction

JABAWS (Troshin *et al.*, 2011) is a system developed to simplify distributed access to bioinformatics command line programs. It consists of a server that can be locally installed or run as a virtual machine, and a client that provides access to supported services *via* an application programming interface (API) or command line (CLI). Here we describe new developments introduced in JABAWS 2.2.

JABAWS 1.0 allowed deployment of web services for five commonly used multiple sequence alignment (MSA) algorithms, and came complete with precompiled binaries, sources and compilation scripts. JABAWS 2.2 provides updated versions of the original JABAWS: MSA tools, and adds three additional MSA methods: Clustal Omega (Sievers *et al.*, 2014); MSAprobs (Liu *et al.*, 2010); and GLprobs (Ye *et al.*, 2015). JABAWS 2 further provides new services for: (i) Protein disorder prediction: DisEMBL (Linding *et al.*, 2003a); IUPred (Dosztányi *et al.*, 2005); GlobPlot (Linding *et al.*, 2003b); and JRonn (unpublished work by Troshin and Barton, 2011), which is a Java implementation of Ronn (Yang *et al.*, 2005); (ii) Conservation analysis: 17 measures of protein conservation by Valdar (Valdar, 2002), and the SMERFS algorithm (Manning *et al.*, 2008) for predicting protein functional sites, provided by the AACon package (Golicz, A., Troshin, P. V., Martin, D., Madeira, F., Procter, J. B. & Barton, G. J. 2017, paper in preparation.), and 3) RNA secondary structure prediction by the RNAalifold program (Bernhart *et al.*, 2008).

JABAWS 2.2 setup and installation has been simplified, and a new execution statistics collector records service usage. Several utility web services are also provided, and the web interface, which has been redesigned and improved, employs these to provide service status information. Metadata and registry services allow JABAWS 2.2 clients to discover supported web services, their execution limits and parameters, and their operational status.

## 2 Implementation and availability

The public JABAWS 2.2 server at the University of Dundee (http://www.compbio.dundee.ac.uk/jabaws) allows alignments and sequence analyses of up to 1000 sequences with up to 1000 residues. Version 2.2 was deployed in August 2017, and features updated third-party programs and a new website. The popular sequence analysis suite, Jalview (Waterhouse *et al.*, 2009) accesses this public JABAWS server by default, so backwards compatibility is essential. JABAWS 2.2 web services are compatible with older JABAWS clients (versions 1.0, 2.0.1 or 2.1). However, the JABAWS 2.2 client is required to access the latest services and features.

The website provides three distributions suitable for most use-cases: (i) the Web Application Archive (WAR); (ii) the Virtual Appliance (VA); and (iii) The Command Line Interface (CLI) Client. As with JABAWS 1.0, the first two distributions allow installation on the user’s laptop/desktop or computer cluster. Local installation permits secure access to services without the use of public networks as well as larger alignments and analyses to be performed. The WAR package can be deployed on any Mac or Linux machine, provided that Java (Version 7 or above) and Apache Tomcat (http://tomcat.apache.org/, version 8.5 or above) are installed. The web application is easy to setup, to scale up and to maintain, and is suited to those wishing to leverage their own infrastructure. Multiple JABAWS applications may also be deployed, allowing custom execution configurations. The JABAWS VA is a simpler solution for those who need a private server. The VA is distributed as a pre-configured Turnkey Linux (https://www.turnkeylinux.org/) virtual machine that can be deployed with VMware (http://www.vmware.com/products) or a free alternative such as VirtualBox (https://www.virtualbox.org). The JABAWS VA is also configured to apply patches automatically with the latest security updates. Both WAR and VA provide access to the same features as the public server, including the execution statistics collector, web services testing and monitoring services, and third-party bioinformatics tools. The final JABAWS distribution is the CLI client, which allows sequence analysis via JABAWS web services to be scripted. Java Version 7 or above is required to run the client, which can access both publicly accessible (University of Dundee) and private JABAWS 2.2 servers (i.e. a local VA or WAR installation). Additional details on how to install and use the three JABAWS distributions are provided in the documentation pages.

One key objective in JABAWS 2.2 was to simplify deployment. Once installed, the WAR and VA provide a suite of tools to check the health of the web services. These include a web services status page, where JABAWS server configuration, status and the details of each service can be reviewed. Internally, the web services status page employs a registry service to discover and evaluate the status and functionality of each service. This helps to eliminate any discrepancies between the two, and the same tests are available from the JABAWS CLI client. Once up and running, the new JABAWS 2.2 web services registry provides a categorized view of the web services running on a particular JABAWS 2.2 instance. Individual services can then be queried for meta-information such as execution limits and documentation URLs. JABAWS 2.2 also allows easy access to usage statistics, for users and administrators. The statistics collector included in JABAWS 2.2 employs a ‘crawler’ for collecting and recording the web services execution. This feature gives a detailed breakdown of the usage of each service. It helps to detect potential performance issues and aids with web services troubleshooting, which is especially useful for system administrators. Once usage statistics have been collected they are recorded in a database, and if desired, job logs and results files can then be safely deleted. System administrators supporting JABAWS servers over long periods of time will find this feature useful as it reduces the administration burden and saves disk space. JABAWS statistics record start and finish time for each job, where the job was executed (e.g. cluster or locally) and the size of the output file. For security and data protection reasons, access to detailed JABAWS statistics is limited to the system administrators only. Users who do not require this service can switch it off.

## 3 Summary

JABAWS is open source and free software. The public JABAWS server is supported by the high-performance cluster of the University of Dundee, School of Life Sciences and is an Elixir-UK Tier 1 Resource (https://www.elixir-uk.org/), as part of the Dundee Resources.
